# Prolonged Hematogone Expansion Is Associated with Better Outcomes in Allogeneic Hematopoietic Stem Cell Transplantation Recipients

**DOI:** 10.3390/hematolrep17050046

**Published:** 2025-09-10

**Authors:** Bianca Serio, Danilo De Novellis, Marisa Gorrese, Angela Bertolini, Paola Manzo, Francesca Picone, Anna Maria Della Corte, Rossella Marcucci, Denise Morini, Michela Rizzo, Roberto Guariglia, Serena Luponio, Pasqualina Scala, Francesco Verdesca, Anna Maria Sessa, Francesca Velino, Martina De Leucio, Maddalena Langella, Valentina Giudice, Carmine Selleri

**Affiliations:** 1 Hematology and Transplant Center, University Hospital “San Giovanni di Dio e Ruggi d’Aragona”, 84131 Salerno, Italy; 2 Department of Medicine, Surgery, and Dentistry, University of Salerno, 84081 Baronissi, Italy; pamanzo@unisa.it

**Keywords:** hematogones, hematopoietic stem cell transplantation, flow cytometry, biomarker

## Abstract

**Background/Objectives**: Hematogones, B cell precursors, are considered a clock of bone marrow reconstitution after chemotherapy and hematopoietic stem cell transplantation (HSCT). **Methods**: In this retrospective observational monocentric study, we investigated the prognostic role of hematogone expansion after allogeneic HSCT and its association with clinical and molecular features. **Results**: Using a cut-off value of 0.1%, hematogones were detected in 60% of patients at the first re-evaluation after HSCT (median, 2.4%; range, 0.2–9.0%) and in 63% of subjects at the most recent evaluation (MRR) (median, 1.4%; range, 0.1–5.1%). In particular, prolonged hematogone expansion was associated with longer overall survival (*p* = 0.0043) and relapse-free survival (*p* = 0.0002). No associations were described between hematogone frequency and stem cell sources or acute or chronic graft versus host disease incidence. **Conclusions**: In conclusion, our results confirmed that hematogones mirrored bone marrow fitness and reconstitution ability; thus, they could be used as a prognostic marker of HSCT outcomes.

## 1. Introduction

Allo-HSCT remains the only curative therapeutic approach for several hematological malignancies and consists of the infusion of donor hematopoietic stem cells (HSCs) following a myeloablative conditioning regimen to restore normal hematopoiesis and/or treat hematological malignancies [1]. CD34^+^ HSCs can be sourced from bone marrow (BM), peripheral blood, or umbilical cord blood, with differences in clinical outcomes, including engraftment time and the incidence of transplant-related complications, such as Graft versus Host Disease (GvHD) [2]. Beneficial effects of HSCT are related to myeloablation post-conditioning regimens and immune responses against leukemic cells driven by donor lymphocytes (also termed graft versus leukemia) [3,4]. However, HSCT outcome is negatively influenced by early and late complications, caused by the excessive myelotoxicity of conditioning drugs and by immunosuppressive therapy used for GvHD prophylaxis [3]. For example, immunosuppression can impair lymphocyte reconstitution, leading to the loss of self-tolerance and proliferation of autoreactive lymphocytes, especially when T regulatory cell recovery is delayed [5]. Other transplant-related complications are mucositis, increases in liver enzymes, hemorrhagic cystitis mainly caused by BKV reactivation, infectious complications due to severe neutropenia, and viral reactivations. Late complications include autoimmune diseases and endocrinopathies [6]. Risk factors influencing relapses and non-relapse mortality (NRM) include donor type (e.g., matched related, matched unrelated, or haploidentical), donor age, gender, Human Leukocyte Antigen (HLA) matching, disease type, and cytomegalovirus (CMV) serostatus [7].

Hematogones were first identified in 1937 as “lymphoid-appearing cells” in the BM of children, and their origins and functions were unknown until recent years, where they have been recognized as physiological B cell precursors, characterized by highly condensed, uniform nuclear chromatin, scant cytoplasm, and diameters ranging from 10 to 20 μm [8,9,10]. Under optical microscopy, immature hematogones are indistinguishable from ALL blasts; however, they can be more precisely identified and classified by multiparametric flow cytometry immunophenotyping, as they are CD19^+^CD45^dim^CD22^dim^CD38^bright^CD43^+^CD10^+^CD34^−^ and TdT^+^ in their more immature stages [10,11,12]. Under physiological conditions, hematogones are present in the BM, although at low frequencies (~5% of total bone marrow-mononuclear cells [BM-MNCs]) [12,13]. However, their number can increase during various clinical conditions, including autoimmune diseases, congenital BM disorders, immunosuppression, CMV infection, and certain neoplastic diseases, such as lymphomas. Hematogones are also frequently observed in highly regenerative BM, like following chemotherapy or allo-HSCT [10], where their expansion correlates with donor HSC repopulation capacity and immune reconstitution, contributing to favorable outcomes [14,15,16]. Indeed, hematogone levels >5% of total BM-MNCs at engraftment are associated with prolonged overall survival (OS) and relapse-free survival (RFS) [17].

In this monocentric observational retrospective study, we aimed to evaluate the prognostic role of hematogone expansion after allo-HSCT, and its association with multiple clinical, phenotypical, molecular, and donor/recipient variables.

## 2. Materials and Methods

### 2.1. Study Cohort

A total of 60 consecutive patients with hematologic malignancies who underwent allo-HSCT outside clinical trials between 2016 and 2022 at the Hematology and Transplant Center, University Hospital “San Giovanni di Dio e Ruggi d’Aragona”, Salerno, Italy, were included in this retrospective study. Inclusion criteria were age ≥18 years; and allo-HSCT indication for any hematological malignancy, according to current guidelines [18]. This study was conducted in accordance with the Declaration of Helsinki and protocols approved by our Ethics Committee “Campania Sud,” Brusciano, Naples, Italy (prot./SCCE no. 24988). All patients provided written informed consent.

### 2.2. Data Collection

Collected data were categorized into four groups: (1) patient-related: age, sex, CMV serostatus, the presence of hematogones, and clinical parameters including complete blood counts (WBC, Hb, and platelets), LDH, and creatinine levels before transplant and at the MRR; (2) disease-related: the type of hematological malignancy, comorbidities, presence of pathogenic somatic mutations or variant of unknown significance (VUS), and cytogenetic abnormalities; (3) HSCT-related: conditioning regimen (myeloablative conditioning (MAC) vs. reduced intensity conditioning (RIC)), HSC source (peripheral blood vs. BM), the number of hematopoietic stem cells infused, engraftment time, full-donor chimerism time, and clinical parameters (blood counts, lactate dehydrogenase (LDH), and creatinine at HSCT); and (4) donor-related factors: age, sex, CMV serostatus, donor relationship (related vs. unrelated), HLA compatibility (matched vs. mismatched), and AB0 compatibility (matched vs. mismatched).

### 2.3. Flow Cytometry

Flow cytometry immunophenotyping was conducted as per our Institution’s clinical practice guidelines at 1 month before transplant, before +100 days from transplant, at 6 months for 2 years, and then yearly for up to 5 years, unless clinical and laboratory signs of disease relapses were present. Hematogone immunophenotyping was carried out by multiparametric flow cytometry on whole BM samples, using the following antibodies for BM cell population characterization: CD3, CD7, CD5, CD19, CD34, CD16, CD11b, CD13, CD14, CD56, CD45, CD33, HLA-DR, CD117, SmIg-kappa, and SmIg-lambda (all from Beckman Coulter, Brea, CA, United States). In brief, 50 μL of blood were stained with conjugated antibodies according to manufacturers’ instructions, and after 20 min incubation at room temperature, red blood cell lysis was performed with IO Test Lysing Solution (Beckman Coulter). Subsequently, samples were washed twice with PBS (IsoFlow Sheath Fluid, Beckman Coulter), and resuspended in 500 μL of PBS for acquisition on a Navios, Navios/EX, or DxFlex cytometer (Beckman Coulter). Instrument daily quality control check was carried out using Flow-Check Pro Fluorospheres (Beckman Coulter) and external quality control by UK NEQAS for Leucocyte Immunophenotyping. Compensation was monthly checked by a Beckman Coulter’s Specialist using a flow-set and compensation kit (Beckman Coulter). Samples were run using the same PMT voltages, and at least 500,000 events were recorded. Post-acquisition analysis was performed using Navios Software v1.3, Navios EX Software v2.0, or Kaluza Analysis Flow Cytometry Software v2.1.1 (Beckman Coulter).

**Figure 1:**
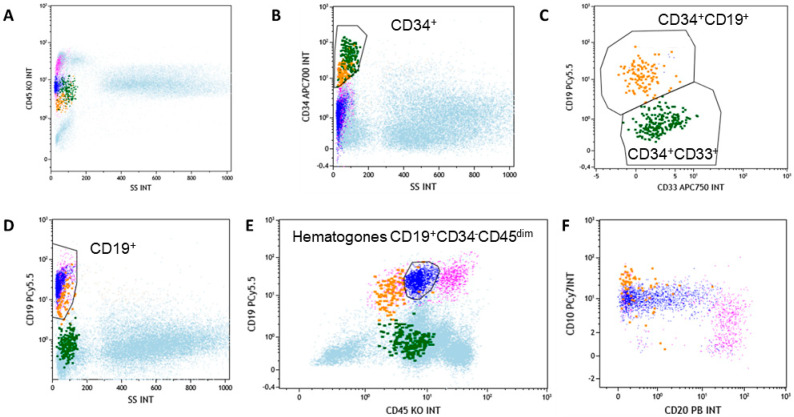
Gating strategy for hematogone identification by flow cytometry. (**A**) Cells displayed using linear parameter (side scatter) and CD45 expression. (**B**) CD34^+^ cells (green and orange dots) are identified based on linear parameters and CD34 expression, and (**C**) further analyzed for CD19 (orange dots) and CD33 expression to exclude CD34^+^CD33^+^ precursors (green dots). (**D**) On CD19^+^ cells, three populations were identified: (**E**) CD34^+^CD19^+^CD45^dim^ precursors, CD34^-^CD19^+^CD45^dim^ hematogones (blue dots), and mature CD34^−^CD19^+^CD45^+^ lymphocytes. (**F**) Hematogones were next characterized for CD10 and CD20 expression.

First, cells were gated based on linear parameters (FSC-A vs. SSC-A), and CD45 expression, and lymphocytes, monocytes, granulocytes, and immature cells were gated (Figure 1). Next, CD34^+^ cells were identified based on linear parameters and CD34 expression and further analyzed for CD19 and CD33 expression to exclude CD34^+^CD33^+^ precursors. On CD19^+^ cells, three populations were identified: CD34^+^CD19^+^CD45^dim^ precursors, CD34^-^CD19^+^CD45^dim^ hematogones, and mature CD34^−^CD19^+^CD45^+^ lymphocytes. Hematogones were next characterized for CD10 and CD20 expression, showing CD10^+/dim^ positivity with variable CD20 expression.

### 2.4. Molecular Biology Analysis

HLA typing was performed by initial screening using a Sequence Specific Primers PCR and confirmed by NGS. *FLT3* and *NPM1* mutations were detected by PCR and RT-PCR using certified kits, as per routine clinical practice. For somatic mutations and VUS, NGS analysis was performed using a SOPHIA Genetics Myeloid Solution panel (Sophia Genetics, Saint Sulpice, Switzerland), covering 30 genes, and analyzed using SophiaDDM software (v4-4.6.2; Sophia Genetics) for alignment and analysis. Variant pathogenicity was defined according to COSMIC and ClinVar databases, while those not reported in these databases were classified as pathogenic based on *in silico* predictions using SIFT, PolyPhen2, or FATHMM. For VUS, ClinVar and VarSome databases were also employed for variant calling.

### 2.5. Statistical Analysis

Data were collected in spreadsheets and analyzed using Prism (v.8.3.0; GraphPad software, San Diego, CA, USA) and SPSS (v. 25; IBM). Differences between groups were assessed by Chi-square, Fisher’s, Wilcoxon signed-rank, or unpaired two-tailed t-tests. Pearson analysis was used for continuous variable correlations. Kaplan–Meyer, log-rank, and Breslow tests were employed for survival analysis, and univariate and multivariate Cox regression models were used to examine effects (HR) of independent variables on survival and hematogone emergence, using a backward stepwise model. All studied variables were included in both univariate and multivariate analysis; however, because of the small number of subjects per group and high biological variability, results from multivariate analysis were not always measurable (shown as “-” in the tables). For data cut-off value identification, patients were divided according to clinical outcomes (alive/dead) and percentage of hematogones before transplant was used for ROC analysis between groups, showing an AUC of 0.7214 (*p* = 0.0118). A cut-off of 0.1% of total nucleated cells was used to divide patients based on the presence or absence of hematogones, as this value displayed good sensitivity (57.1%; 95% CI, 40.9–72.02%) and specificity (68.8%; 95% CI, 44.4–85.8%; likelihood ratio, 1.829). A *p* value of < 0.05 was considered statistically significant.

## 3. Results

### 3.1. Patients’ Characteristics at Allo-HSCT

Recipients’ and donors’ characteristics at the time of allo-HSCT are summarized in Table 1.

The majority of patients in our cohort received a diagnosis of AML (n = 36, 60%) and ALL (n = 11; 18%), while the remaining subjects suffered from MDS (n = 6; 4%) or MM (n = 6; 4%). Cytogenetic abnormalities were observed in 28% of patients, while pathogenic somatic mutations were observed in 15% of subjects. In AML cases, *FLT3* and *NPM1* mutations were documented in 19% of patients each. Comorbidities affected 24 subjects (40%) and included hypercholesterolemia/dyslipidemia (n = 6), diabetes (n = 3), obesity with moderate liver steatosis (n = 1), Crohn’s disease (n = 1), gastritis (n = 2), chronic obstructive pulmonary disease (n = 1), hypertension (17%), and other conditions (23%). The median number of infused CD34^+^ cells was 4.61 × 10^3^/kg (range, 0.58–10.00 cells × 10^3^/kg). Median times to neutrophil engraftment and full donor chimerism were 15 days (range, 9–36 days) and 71 days (range, 30–114 days), respectively. At the latest follow-up, 53% of patients were in complete remission, 5% had disease relapsed, and 42% died. Of those, 60% of deaths (15 out of 25 cases) were associated with transplant-related complications, such as GvHD, infections, and venous occlusive disease, while in the remaining cases, causes were not related to transplant.

Multiparametric flow cytometry was used to detect hematogones before transplantation (initial evaluation), post-HSCT (median time to first re-evaluation, 104 days; range, 35–463 days), and at the MRR (median time to MRR, 522 days; range, 66–2282 days). At the first re-evaluation, hematogones were present in 60% of patients, with median levels of 2.4% (range, 0.2–9.0%), while at the MRR, hematogones were detected in 63% of cases, with median levels of 1.4% (range, 0.1–5.1%).

### 3.2. Impact of Hematogone Expansion on Survival After Allo-HSCT

**Figure 2:**
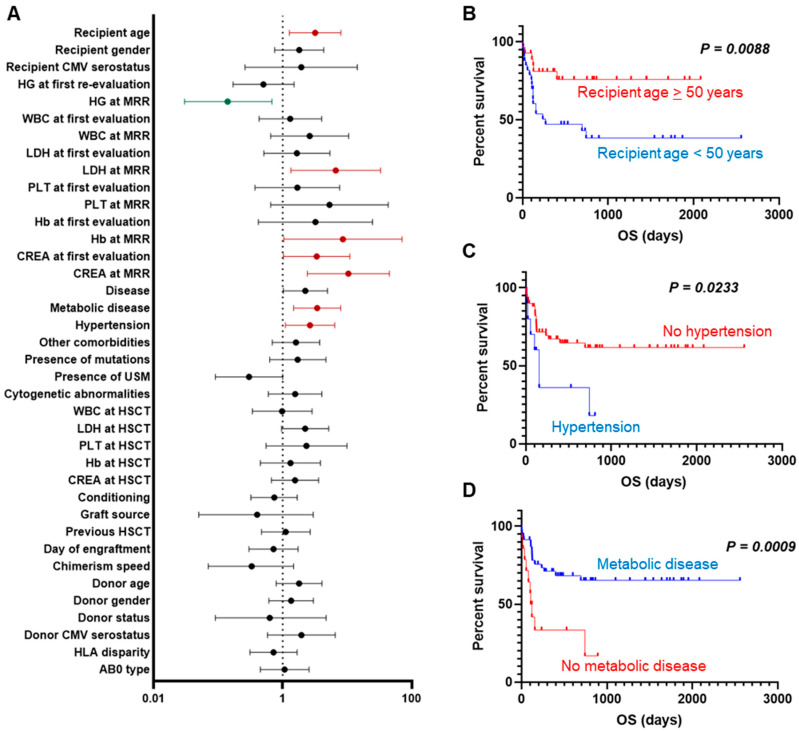
Factors influencing OS after hematopoietic stem cell transplantation. (**A**) Univariate Cox regression analysis on OS. Patients were divided based on various clinical characteristics, such as age (**B**), presence of hypertension (**C**), or metabolic disease (**D**), and OS rates were compared between groups by Log-rank test.

To investigate the impact of various clinical variables on OS in patients with the presence of hematogones at the MRR, univariate and multivariate regression analyses were performed (Table 2 and Figure 2A). Several factors showed a significant association with OS by univariate analysis, including recipient age (<50 or >50 years; *p* = 0.013), reduced LDH levels at the MRR (*p* = 0.020), increased Hb levels at the MRR (*p* = 0.046), creatinine at the first re-evaluation (*p* = 0.044) and MRR (*p* = 0.002), disease type (AML vs. other; *p* = 0.046), hypertension (*p* = 0.030), and the presence of metabolic comorbidities (*p* = 0.004) (Figure 2B–D). Interestingly, the presence of hematogones at the MRR emerged as the sole protective factor for survival (*p* = 0.015), also confirmed by multivariate analysis (*p* = 0.024). Median time to neutrophil engraftment after allo-HSCT was 15 days (range, 9–36 days) and was related to higher hematogone concentration (r = −0.3410; *p* < 0.05), although no significant differences in OS were observed when divided into fast- and slow-engrafting groups.

**Figure 3:**
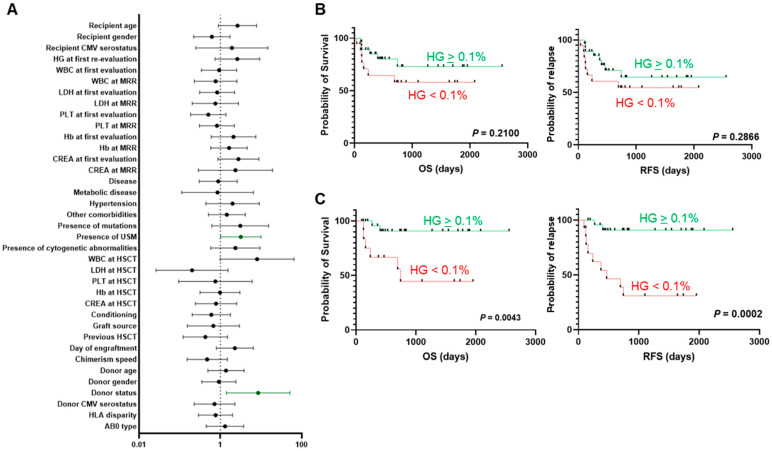
Influence of hematogone frequency on clinical outcomes. (**A**) Univariate regression analysis (dependent variable = HG presence at the MRR). Patients were divided based on HG frequency <0.1% at first re-evaluation (**B**) or at the MRR (**C**), and OS and RFS rates were compared between groups by Log-rank test.

Next, patients were divided into two groups based on a hematogone cut-off of >0.1%, and the impact on OS and RFS was evaluated at the first re-evaluation and MRR (Figure 3). In particular, the presence of bone marrow hematogones at the first re-evaluation with a median percentage of 2.4% (range, 0.02–9.00%) was not significantly associated with prolonged OS and RFS (*p* = 0.2100 and *p* = 0.2866, respectively; Figure 3B), while their presence at the MRR (median hematogone frequency, 1.43%; range, 0.01–5.10%) was linked to a significant increase in OS and RFS (*p* < 0.01 and *p* < 0.001, respectively; Figure 3C). Moreover, higher bone marrow hematogone frequency at the first re-evaluation was associated with hematological complete response after allo-HSCT compared to those frequencies observed in patients who died or relapsed (median hematogone percentage, 1.96% [range, 0–9%] vs. 0.6% [range, 0–3.5%], complete remission vs. disease relapse or death; *p* = 0.052).

### 3.3. Factors Influencing Hematogone Expansion

Finally, the impact of various factors on hematogone expansion was investigated by univariate and multivariate Cox regression analyses (Table 3 and Figure 3A). By univariate analysis, the presence of variants of uncertain significance and donor status (related vs. unrelated) were significantly associated with hematogone expansion at the MRR (*p* = 0.048 and *p* = 0.020, respectively), also confirmed by multivariate analysis (*p* = 0.009). In particular, patients carrying variants of uncertain significance displayed higher median hematogone frequencies compared to those without these genomic alterations (median frequency, 1.7% vs. 0.6%; range, 0–4.7% vs. 0–5.1%, respectively; *p* = 0.042).

## 4. Discussion

Disease relapse after HSCT is still a major complication in hematological malignancies, negatively affecting prognosis, thus the identification of prognostic markers for the early detection of disease progression and relapse after transplantation is of critical importance, especially in the real-world clinical setting. Hematogones CD19^+^CD34^−^CD45^dim^ B lymphoid precursors are transiently increased during hematological recovery after intensive chemotherapy and bone marrow transplant [10]. Historically, hematogones have referred to various terms, such as blasts, marrow precursor cells, post-therapeutic stem cells, lymphocytes, terminal deoxynucleotidyl transferase-positive cells, and common acute lymphoblastic leukemia antigen-positive cells [10,11,12]. Only in recent years, with the advent of multiparametric flow cytometry, has specific hematogone immunophenotyping been described [19,20], favoring their distinction from other cells. In this retrospective observational real-world study, the potential prognostic role of hematogone recovery following allo-HSCT was investigated in a cohort of 60 consecutive patients who underwent transplantation for various hematological malignancies.

Hematogones are considered indicators of donor stem cell proliferative capacity in repopulating the recipient’s bone marrow niche; however, their clinical significance remains unclear [10]. In AML, their presence at the time of hematological recovery after intensive chemotherapy has been associated with improved RFS and OS compared to subjects without hematogone expansion. Furthermore, detectable marrow hematogones independently predict RFS (hazard ratio = 0.61, 95% CI: 0.42–0.89, *p* = 0.012) and OS (hazard ratio = 0.59, 95% CI: 0.38–0.92, *p* = 0.019) [21]. Indeed, hematogone expansion has also been linked to complete remission and lower WT1 levels after induction chemotherapy [22,23].

While evidence of clinical significance of hematogone expansion after allogenic HSCT is limited, previous studies suggest that their presence could represent an independent prognostic factor of OS, regardless of donor source [13,24,25]. In our study, we longitudinally monitored bone marrow hematogone frequency in a cohort of allo-HSCT recipients, and we found that hematogone recovery was significantly associated with prolonged survival, especially in those subjects with sustained hematogone expansion mirroring long-term regenerative capacity of donor stem cells. This correlation was documented in all types of HSC source, in contrast with previous results reporting that hematogone emergency is more frequent in cord blood and peripheral blood stem cell transplantation compared to bone marrow transplant [14,26].

Hematogone emergency after chemotherapy or HSCT has been associated with leukemic cell eradication and rapid immune reconstitution [17]. After transplantation, increased hematogone frequencies are associated with higher absolute peripheral lymphocyte counts and serum IgG levels, reflecting the immune reconstitution process, especially within 100 days from HSCT [14,17,27,28]. In addition, corticosteroid use does not affect hematogone recovery, as these cells could normally expand after steroid treatment without impacting OS and the incidence of acute GvHD [14]. To further explore factors influencing hematogone expansion in the bone marrow niche after allo-HSCT, we performed univariate and multivariate analysis. Interestingly, the presence of VUS was associated with increased hematogone frequencies at the MRR in multivariate analysis. Although the precise role of these mutations remains unclear, they could reflect clonal hematopoiesis and genetic heterogeneity of HSC pool, regardless of the known pathogenic variant [29]. Consequently, donor-derived HSC clonal heterogeneity and fitness might influence stem cell proliferation and maturation, potentially contributing to effective hematopoiesis or graft failure, based on the functional impact of these variants [30].

Multiparametric flow cytometry immunophenotyping is more sensitive and specific for the identification of cell populations compared to optical microscopy, as previously documented for leukemic cells and hematogones [24,26,31]. For hematogone detection, pre-analytical variables, such as BM hemodilution during specimen collection must be carefully considered, as this can negatively influence their frequency [14]. Indeed, BM threshold levels widely vary across studies, ranging from >0% to 5% [24,25,26,27]. In particular, previous meta-analysis has indicated that hematogones >0.01% are associated with a significantly better leukemia-free and OS, while in transplant recipients, a percentage >5% at one month from transplantation is related with a significantly longer OS and a lower risk of acute GvHD [21,32]. In our study, a threshold of 0.1% has been proposed based on ROC analysis, also according to flow cytometry sensitivity; however, no significant differences in OS using this cut-off have been described [14]. In contrast, we found that hematogone recovery within the first 100 days from transplant was not associated with prognosis when using the 0.1% threshold, while sustained hematogone expansion over time was linked to improved outcomes, including prolonged OS and RFS. Previous studies have indicated differences in hematogone percentages and clinical outcomes based on underlying diseases [21,33]. For example, in lymphomas and non-neoplastic cytopenias, hematogones could be >5%, while they are greatly reduced in myelodysplastic syndromes and myeloproliferative neoplasms [34]. In AML and ALL, hematogone increase after chemotherapy has been associated with disease relapse [21,34,35], while after allogeneic stem cell transplantation, their expansion mimics bone marrow reconstitution [26,28]. Similarly, hematogone expansion is also a frequent condition after autologous stem cell transplantation, as described in multiple myeloma patients [33]. In this condition, subjects with hematogone increase after autologous transplantation show a higher risk of disease progression or relapse and worse outcomes, and the ratio between hematogone percentage and the number of stem cells infused could better define prognosis in multiple myeloma patients who undergo autologous HSCT [33]. Indeed, the presence of hematogones in apheresis products is associated with better outcomes, especially shorter neutrophil engraftment time, in both allogeneic and autologous HSCT recipients [36]. In our study, we showed that hematogone expansion, defined using a lower cut-off of 0.1%, could predict clinical outcomes in transplant recipients regardless of underlying diseases.

Our study has several limitations: (i) retrospective design; (ii) the lack of randomization to control potential confounding variables; (iii) the clinical heterogeneity of enrolled patients, including various primary hematological malignancies and transplant types; (iv) variability in conditioning regimens for allogenic HSCT, which could affect marrow reconstitution; (v) additional analysis for investigation of immune reconstitution after transplantation; and (vi) the small number of subjects for each clinical entity or transplant type, which could increase the risk of type II errors (false negatives), especially when clinically heterogenous conditions are investigated, such as disease types, genetic features, and risk stratification groups. This wide biological variability is due to patient-specific genomic and phenotypic signatures that increase type II errors if the subcategory sample size is not adequate; therefore, we could not exclude additional statistically significant differences between groups.

## 5. Conclusions

In conclusion, our data support the evidence that hematogone recovery and sustained emergence after allo-HSCT are independent prognostic factors, independently from HSC sources and recipient characteristics. Moreover, prolonged hematogone expansion could reflect an active proliferating niche, leukemic cell eradication, immune reconstitution, and increased immunosurveillance, eventually leading to better OS and RFS, also in a real-world setting. However, larger prospective studies are warranted to confirm these results and further validate the prognostic value of hematogone monitoring following allogeneic transplantation.

## Figures and Tables

**Table 1:** Patients’ and donors’ characteristics at transplant.

	Recipient N = 60	Donor N = 60
Median age, years (range)	50 (19–71)	44 (17–69)
Gender (M/F), n (%)	M 37 (62)	M 30 (50)
CMV positivity, n (%)	56 (93)	47 (78)
HLA-mismatch		
HLA-identical, n (%)		37 (62)
Haplo-identical, n (%)		23 (38)
AB0 homogroup, n (%)		18 (30)
Donor status		
MRD, n (%)		56 (93%)
MUD, n (%)		4 (7%)
Comorbidities, n (%)	24 (40)	
Metabolic, n (%)	13 (22)	
Hypertension, n (%)	10 (17)	
Others, n (%)	14 (23)	
Diagnosis		
AML	36 (60)	
ALL	11 (18)	
MDS	6 (10)	
MM	6 (10)	
T-cell NHL	1 (2)	
Cytogenetics abnormalities, n (%)	17 (28)	
Pathogenic/VUS mutations, n (%)	9 (15)/17 (28)	
*FLT3* mutated	7 (19)	
*NPM1* mutated	7 (19)	
WT1 upregulation	19 (53)	
Median ANC, cells/µL (range)	2246 (20–14,170)	
Median platelets, cells/µL (range)	85,703 (1000–298,000)	
Median LDH U/L (range)	523 (200–3326)	
Conditioning		
RIC, n (%)	36 (60)	
MAC, n (%)	24 (40)	
HSC source		
PBSCs, n (%)	55 (92)	
BMSCs, n (%)	5 (8)	
Median CD34^+^ cells × 10^3^/Kg infused (range)	4.61 (0.58–10.00)	
Median days to engraftment (range)	15 (9–36)	
Median days to full chimerism (range)	71 (30–114)	
Median follow-up, months	21	

Abbreviations. HLA, human leukocyte antigen; MRD, matched-related donor; MUD, matched-unrelated donor; AML, acute myeloid leukemia; ALL, acute lymphoblastic leukemia; MDS, myelodysplastic syndrome; MM, multiple myeloma; NHL, non-Hodgkin lymphomas; WT1, Wilm’s tumor 1; ANC, absolute neutrophil count; RIC, reduced intensity conditioning; MAC, myeloablative conditioning; HSCs, hematopoietic stem cells; PBSCs, peripheral blood stem cells; BMSCs, bone marrow stem cells.

**Table 2:** Univariate and multivariate analysis on OS with the presence of hematogones at the MRR.

Dependent Variable = OS		Univariate		Multivariate	
**Patient-Related Factors**	**Comparison**	**Odds** **(95% CI)**	***p* Value**	**Odds** **(95% CI)**	***p* Value**
Recipient age	<50 vs. ≥50 years	2.141 (0.97–4.74)	0.060	-	-
Recipient gender	Male vs. female	2.1 (0.89–4.95)	0.090	-	-
Recipient CMV serostatus	Positive vs. negative	2.249 (0.31–16.57)	0.426	-	-
Hematogones at first re-evaluation	Presence vs. absence	0.59 (0.22–1.58)	0.292	-	-
Hematogones at MMR	Presence vs. absence	0.094 (0.02–0.43)	0.002	0.357 (0.05–2.63)	0.019
WBC at first evaluation	Normal vs. out of range	1.348 (0.49–3.72)	0.564	-	-
WBC at MRR	Normal vs. out of range	4.486 (1.30–15.46)	0.017	-	-
LDH at first evaluation	Normal vs. out of range	1.266 (0.46–3.49)	0.649	-	-
LDH at MRR	Normal vs. out of range	5.764 (1.53–21.76)	0.010	-	-
Platelets at first evaluation	Normal vs. out of range	1.323 (0.38–4.66)	0.662	-	-
Platelets at MRR	Normal vs. out of range	6.681 (0.846–52.769)	0.072	-	-
Hb at first evaluation	Normal vs. out of range	1.177 (0.34–4.14)	0.800	-	-
Hb at MRR	Normal vs. out of range	5.329 (1.14–24.99)	0.034	0.739 (0.34–1.62)	0.024
Creatinine at first evaluation	Normal vs. out of range	3.432 (1.19–9.92)	0.023	-	-
Creatinine at MRR	Normal vs. out of range	9.396 (2.63–33.55)	0.001	1.568 (0.71–3.47)	0.019
**Disease-related factors**	**Comparison**	**Odds** **(95% CI)**	***p* Value**	**Odds** **(95% CI)**	***p* Value**
Disease	AML vs. others	1.805 (0.86–3.8)	0.120	-	-
Metabolic disease	Presence vs. absence	4.282 (1.91–9.60)	0.000	-	-
Hypertension	Presence vs. absence	2.299 (0.97–5.43)	0.058	-	-
Other comorbidities	Presence vs. absence	1.659 (0.75–3.69)	0.214	-	-
Mutations	Presence vs. absence	1.92 (0.76–4.86)	0.169	-	-
VUS	Presence vs. absence	0.372 (0.13–1.09)	0.072	-	-
Cytogenetic abnormalities	Presence vs. absence	1.376 (0.57–3.31)	0.477	-	-
WBC at HSCT	Normal vs. out of range	0.868 (0.33–2.28)	0.774	-	-
LDH at HSCT	Normal vs. out of range	2.243 (1.01–4.97)	0.046	-	-
Platelets at HSCT	Normal vs. out of range	1.673 (0.51–5.55)	0.400	-	-
Hb at HSCT	Normal vs. out of range	1.173 (0.45–3.09)	0.746	-	-
Creatinine at HSCT	Normal vs. out of range	1.568 (0.71–3.47)	0.267	-	-
**HSCT-related factors**	**Comparison**	**Odds** **(95% CI)**	***p* Value**	**Odds** **(95% CI)**	***p* Value**
Conditioning	MAC vs. RIC	0.739 (0.34–1.62)	0.449	-	-
Graft source	PB vs. BM	0.357 (0.05–2.63)	0.311	-	-
Previous HSCT	Yes vs. no	0.955 (0.41–2.25)	0.916	-	-
Days to engraftment	<16 vs. ≥16 days	0.487 (0.19–1.24)	0.133	-	-
Chimerism time	<71 vs. ≥71 days	0.253 (0.06–1.12)	0.070	-	-
**Donor-related factors**	**Comparison**	**Odds** **(95% CI)**	***p* Value**	**Odds** **(95% CI)**	***p* Value**
Donor age	<44 vs. ≥44 years	1.805 (0.83–3.91)	0.135	-	-
Donor gender	Male vs. female	1.232 (0.59–2.59)	0.582	-	-
Donor status	MRD vs. MUD	0.584 (0.08–4.32)	0.598	-	-
Donor CMV serostatus	Positive vs. negative	1.588 (0.55–4.59)	0.394	-	-
HLA mismatch	Match vs. mismatch	0.605 (0.27–1.38)	0.231	-	-
AB0 type	Match vs. mismatch	1.021 (0.45–2.32)	0.960	-	-

**Table 3:** Univariate e multivariate analysis on hematogone emergency.

Dependent Variable = Hematogone Emergency		Univariate		Multivariate	
**Patient-Related Factors**	**Comparison**	**Odds** **(95% CI)**	***p* Value**	**Odds** **(95% CI)**	***p* Value**
Recipient age	<50 vs. ≥50 years	2.641 (0.89–7.78)	0.078	-	-
Recipient gender	Male vs. female	0.616 (0.22–1.72)	0.356	-	-
Recipient CMV serostatus	Positive vs. negative	1.922 (0.25–14.75)	0.530	-	-
Hematogones at first re-evaluation	Presence vs. absence	2.607 (0.74–9.22)	0.137	-	-
WBC at first evaluation	Normal vs. out of range	0.925 (0.34–2.50)	0.878	-	-
WBC at MRR	Normal vs. out of range	0.765 (0.23–2.56)	0.664	-	-
LDH at first evaluation	Normal vs. out of range	0.832 (0.31–2.42)	0.716	-	-
LDH at MRR	Normal vs. out of range	0.752 (0.20–2.79)	0.670	-	-
Platelets at first evaluation	Normal vs. out of range	0.505 (0.19–1.38)	0.181	-	-
Platelets at MRR	Normal vs. out of range	0.821 (0.31–2.21)	0.695	-	-
Hb at first evaluation	Normal vs. out of range	2.104 (0.59–7.44)	0.249	-	-
Hb at MRR	Normal vs. out of range	1.625 (0.58–4.57)	0.357	-	-
Creatinine at first evaluation	Normal vs. out of range	2.768 (0.86–8.88)	0.087	-	-
Creatinine at MRR	Normal vs. out of range	2.348 (0.29–19.19)	0.426		
**Disease-related factors**	**Comparison**	**Odds** **(95% CI)**	***p* Value**	**Odds** **(95% CI)**	***p* Value**
Disease	AML vs. others	0.884 (0.30–2.59)	0.822	-	-
Metabolic disease	Presence vs. absence	0.851 (0.11–6.49)	0.876	-	-
Hypertension	Presence vs. absence	1.987 (0.44–9.02)	0.374	-	-
Other comorbidities	Presence vs. absence	1.439 (0.51–4.11)	0.496	-	-
Mutations	Presence vs. absence	3.074 (0.62–15.35)	0.171	-	-
VUS	Presence vs. absence	3.171 (1.01–9.97)	0.048	4.39 (1.46–13.23)	0.009
Cytogenetic abnormalities	Presence vs. absence	2.34 (0.58–9.46)	0.233	-	-
WBC at HSCT	Normal vs. out of range	7.99 (0.98–65.38)	0.053	-	-
LDH at HSCT	Normal vs. out of range	0.201 (0.03–1.55)	0.123	-	-
Platelets at HSCT	Normal vs. out of range	0.753 (0.09–6.04)	0.789	-	-
Hb at HSCT	Normal vs. out of range	0.978 (0.32–3.02)	0.969	-	-
Creatinine at HSCT	Normal vs. out of range	0.777 (0.24–2.51)	0.674	-	-
**HSCT-related factors**	**Comparison**	**Odds** **(95% CI)**	***p* Value**	**Odds** **(95% CI)**	***p* Value**
Conditioning	MAC vs. RIC	0.6 (0.20–1.78)	0.356	-	-
Graft source	PB vs. BM	0.671 (0.15–2.94)	0.597	-	-
Previous HSCT	Yes vs. no	0.426 (0.12–1.49)	0.184	-	-
Days to engraftment	<16 vs. ≥16 days	2.272 (0.79–6.46)	0.124	-	-
Chimerism time	<71 vs. ≥71 days	0.472 (0.15–1.48)	0.198	-	-
**Donor-related factors**	**Comparison**	**Odds** **(95% CI)**	***p* Value**	**Odds** **(95% CI)**	***p* Value**
Donor age	<44 vs. ≥44 years	1.371 (0.49–3.82)	0.546	-	-
Donor gender	Male vs. female	0.917 (0.35–2.42)	0.861	-	-
Donor status	MRD vs. MUD	8.492 (1.40–51.46)	0.020	2.52 (0.5–13.5)	0.282
Donor CMV serostatus	Positive vs. negative	0.717 (0.23–2.28)	0.572	-	-
HLA disparity	Match vs. mismatch	0.759 (0.29–1.99)	0.576	-	-
AB0 type	Match vs. mismatch	1.301 (0.45–3.76)	0.626	-	-

## Data Availability

Data are available upon request by the authors.

## Abbreviations

The following abbreviations are used in this manuscript:

HSCT	Hematopoietic stem cell transplantation
MRR	Most recent re-evaluation
Allo-HSCT	Allogeneic hematopoietic stem cell transplantation
HSCs	Hematopoietic stem cells
BM	Bone marrow
GvHD	Graft versus host disease relapse
NRM	Non-relapse transplant mortality
HLA	Human leukocyte antigen
CMV	Cytomegalovirus
ALL	Acute lymphoblastic leukemia
BM-MNCs	BM mononuclear cells
OS	Overall survival
RFS	Relapse-free survival
WBC	White blood cells
Hb	Hemoglobin levels
PLT	Platelets
LDH	Lactate dehydrogenase
VUS	Variants of unknown significance
MAC	Myeloablative conditioning
RIC	Reduced-intensity conditioning
PBS	Phosphate-buffered saline
FSC-A	Forward scatter area
SSC-A	Side scatter area
HR	Hazard ratio
AML	Acute myeloid leukemia
MDS	Myelodysplastic syndrome
MM	Multiple myeloma
MRD	Matched-related donor
MUD	Matched-unrelated donor
NHL	Non-Hodgkin lymphoma
FLT3	Fms-related receptor tyrosine kinase 3
NPM1	Nucleophosmin
WT1	Wilm’s tumor 1
ANC	Absolute neutrophil count
PBSCs	Peripheral blood stem cells
BMSCs	Bone marrow stem cells
HG	Hematogones
PB	Peripheral blood
CREA	Creatinine
ROC	Receiver Operating Characteristic
AUC	Area under the curve
NGS	Next-generation sequencing
